# Soret vector for description of multicomponent mixtures

**DOI:** 10.1038/s41598-021-97125-6

**Published:** 2021-09-06

**Authors:** Aliaksandr Mialdun, Mounir Bou-Ali, Valentina Shevtsova

**Affiliations:** 1 MRC, CP165/62, Université Libre de Bruxelles, Av. F.D. Roosevelt, 50, 1050 Brussels, Belgium; 2 Mechanical and Manufacturing Department, Mondragon University, Loramendi 4, 20500 Mondragon, Spain; 3 IKERBASQUE, Basque Foundation for Science, Bilbao, Spain

**Keywords:** Chemical physics, Thermodynamics, Fluids

## Abstract

The Soret effect describes the transport of constituent species in multicomponent mixtures that occurs due to a temperature gradient. This cross-coupling effect of heat and mass transfer has been successfully examined in binary liquid mixtures, while experiments with ternary mixtures are rare as they impose significant difficulties. We introduce a new and innovative concept, *the Soret vector*, for the characterization of Soret driven separation in ternary mixtures. The presentation of the component separation in the vector form offers several advantages: (i) to predict the Soret sign of a ternary mixture from knowledge of the Soret coefficients in binary subsystems; (ii) to control consistency of measured coefficients, this is especially important when results are obtained using different instruments and methods; (iii) to determine in which regions and which components cause the greatest separation; (iv) to identify the regions where the Soret separation is inaccessible for optical techniques or gravitationally unstable. We demonstrate these features by exploring ternary mixtures of different origins: (a) nearly ideal mixture composed by THN–IBB–nC12 when Soret coefficients in binary subsystems ($S_{T}^{bin}$) are positive, (b) non-ideal mixture containing water and ethanol TEG–Wat–EtOH when $S_{T}^{bin}$ are positive and negative and (c) Tol–MeOH–Ch mixture containing demixing zone with positive and negative $S_{T}^{bin}$. Our approach provides a promising systematic framework for the future research of an important and challenging problem of thermodiffusion in multicomponent liquids.

## Introduction

Thermodiffusion (also known as thermal diffusion or Soret effect) refers to a transport mechanism in which temperature gradients cause mass transfer in mixtures. It plays a significant role in systems like petroleum reservoirs^1^, chemical and biological systems^2^, colloids and polymers^3–5^, micro- and nanofluidics^6^, ionic liquids^7^ and many others. The separation degree of the components between hot and cold regions is quantified by the Soret coefficient. The sign of the Soret coefficient of a mixture component indicates whether it diffuses into a hot or cold region. A positive Soret coefficient means that the diffusive component concentrates in the cold region, whereas a negative value means it concentrates in the hot region. In addition, the Soret coefficient of a given mixture can change sign depending on temperature and concentration. Experimental techniques for measuring Soret coefficients in liquid binary mixtures have been developed over the years and are described in detail in recent reviews^8,9^. However, multicomponent liquid mixtures play an important role in many processes ranging from industry to cellular biology. In order to bridge the gap between the well-studied binary and multicomponent mixtures, the emphasis of the latest research is placed on ternary mixtures.

If we denote the mass fraction of the component *i* by $w_i$, i.e., ($w_1+w_2+w_3=1$), then in the ternary mixture the diffusive fluxes of the independent components ($i=1,2$) will be written as

1 $$\begin{aligned}&j_1=-\rho (D_{11}\nabla w_1 + D_{12} \nabla w_2 + D_{T,1}^{\prime } \nabla T), \end{aligned}$$

2 $$\begin{aligned}&j_2=-\rho (D_{21}\nabla w_1 + D_{22} \nabla w_2 + D_{T,2}^{\prime } \nabla T), \end{aligned}$$

where $D_{ik}$ are the Fick diffusion coefficients and $D_{Ti}^{\prime }$ are the thermal diffusion coefficient of the component *i*. In the steady state the diffusion fluxes vanish ($j_i=0$), and the components separation is proportional to the imposed temperature gradient

3 $$\begin{aligned} \Delta w^{st}_i = - S_{T,i}^{\prime } \Delta T, \quad i = 1, 2, \end{aligned}$$

where $S_{T,i}^{\prime }$ is the Soret coefficient. The mass conservation requires that

4 $$\begin{aligned} \sum ^3_{i=1}S_{T,i}^{\prime }=0, \quad \text{ where } \quad S_{T,i}^{\prime }=\sum ^{2}_{k=1}(\mathbf{D^{-1}})_{ik} D_{Tk}^{\prime }, \end{aligned}$$

$\mathbf{(D^{-1}})_{ik}$ denotes an element of the inverse diffusion matrix.

Thus, six unknown quantities, four diffusion and two thermodiffusion coefficients, determine both kinetics and steady-state of the Soret separation. However, from a typical Soret experiment, one can obtain with good accuracy only two Soret coefficients and an average diffusion kinetics^10,11^. Hence, the full diffusion matrix has to be measured independently. The values of diffusion coefficients in ternary mixtures depend on the order of the components as well as on the frame of reference for which the diffusive fluxes are written^12^. Accordingly, the value of the sequential Soret coefficient ($S_{T,i}^{\prime }$, $i = 1,2,3$) is assigned to a specific component. We have adopted a hydrodynamic approach to the numbering of components, which corresponds to a decreasing order of density of pure components $\rho _1>\rho _2>\rho _3.$

Ternary mixtures are in general much less predictable and more prone to perturbations than binaries due to the additional possibility of cross diffusion. Gravitational instabilities with temperature gradients can be both of thermal and compositional origin and may perturb on long time scales even an initially stable stratified liquid in thermodiffusion experiments. Furthermore, if only one Soret coefficient, out of two in ternary mixtures, is negative, it is in some cases impossible to identify the presence of instability, even with the most advanced optical methods. Studies of thermodiffusion in liquid ternary mixtures are currently performed not only in ground laboratories^11,13–16^ but also under microgravity aboard the International Space Station (ISS)^17–22^. Measurements under microgravity conditions serve as a benchmark^23^ because the destabilizing effect of buoyant convection is reduced.

The suggested novel quantity, the Soret vector, sheds light on the organization of the Soret separation in a ternary mixture as a whole. It enables recognizing domains where the separation magnitude is higher or lower, as well as identifying which components drive the separation most, and where. Further, the representation of the separation in a ternary system as a vector field allows an instant visual consistency check of all the data. Identifying of outliers in the continuous vector field is extremely easy, as a mismatch in the vector magnitude and direction is immediately striking. Finally, an interesting opportunity to understand the system behaviour appears when the vector field of the Soret separation is superimposed onto a map of properties such as refractive index or density.

## Results

### Methods

Let us introduce a concept of the Soret vector. We consider that the liquid was kept at mean temperature, $T_{mean}=T_{c}+\Delta T/2$, prior to the Soret separation ($T_{c}$ and $T_{h}$ are the temperature of the cold and hot regions and $\Delta T=T_{h}-T_{c}$). Each state point of the ternary mixture in the composition space $(w_1^0, w_2^0)$ is characterized by two Soret coefficients $(S'_{T,1}, S'_{T,2})$. The developing separation is symmetric with respect to the initial composition $w^0_i$, i.e., at the cold side it takes the value $w_i^c = w_i^0 + S'_{T,i}/2$, while at the hot side it is $w_i^h = w_i^0 - S'_{T,i}/2$. These two points $(w_1^h, w_2^h)$ and $(w_1^c, w_2^c)$, symmetrically located around the state point $(w_1^0, w_2^0)$, do fully characterize the considered Soret separation. For visual clarity of the direction of separation, the half of the vector corresponding to the concentration change due to temperature decrease is coloured blue. The coordinates of the blue part are thus defined as $(w_1^0, w_2^0)$ as the start point, and $(w_1^0+S'_{T,1}/2, w_2^0+S'_{T,2}/2)$ as the end point. In the same manner, the half of the vector corresponding to the concentration change due to temperature increase turns red. Coordinates of this red part are defined as $(w_1^0, w_2^0)$ as the start point, and $(w_1^0-S'_{T,1}/2, w_2^0-S'_{T,2}/2)$ as the end point. The two ends of these two half-vectors, symmetrically located around the mean concentration, do visually show how the concentration will evolve in case of applying a unit temperature difference to the mixture.

**Figure 1:**
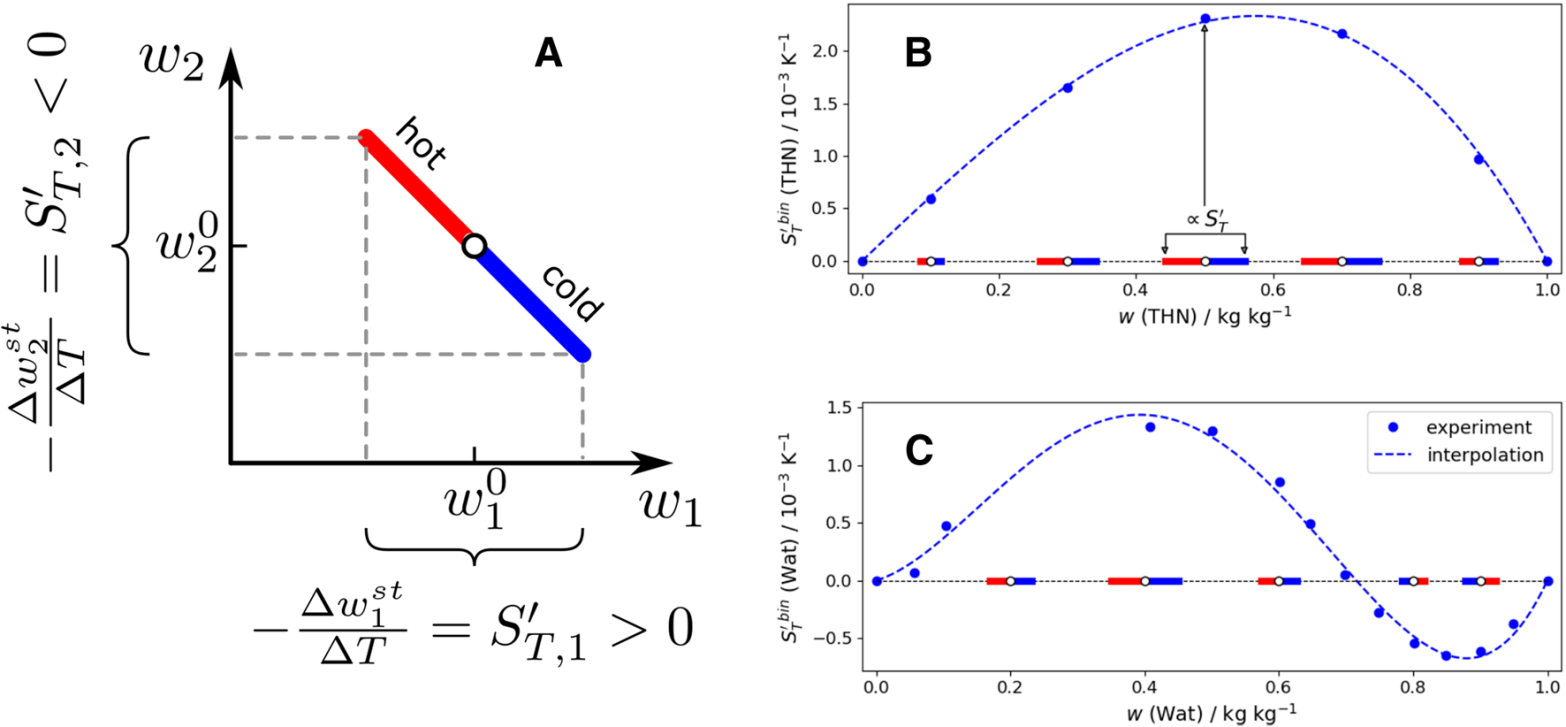
(**A**) Concept of the Soret vector in a ternary mixture. Presentation of the Soret vector over the composition range in binary systems (**B**) the THN–IBB mixture without the sign change of the Soret coefficient and (**C**) the Wat–EtOH mixture with a change in sign. The length of the vectors is shown to scale and is proportional to the value of the Soret coefficients. The Soret coefficients have been taken from literature, THN–IBB (Ref.^24^) and Wat–EtOH (Ref.^25^). The blue dots indicate experimental values while the dashed curves provide a visual guide.

In what follows, the vector connecting these extreme points will be called the “Soret vector”. Figure 1A illustrates the concept of the Soret vector which is fully defined by its components as

5 $$\begin{aligned} \pmb {S'_T} = S'_{T,1} \pmb {e_1} + S'_{T,2} \pmb {e_2}, \end{aligned}$$

where $\pmb {e_i}$ is the unit vector along the axis $w_i$. In the following discussion, we consider that the vector complies with the behavior of a typical physico-chemical property, i.e., varying continuously and smoothly over the concentration space. This means, that its behaviour has to closely resemble the one of a classical vector field.

Before proceeding to the ternary space we consider the vector behaviour in binary systems. Two common examples illustrate binary mixtures without and with a change in the sign of the Soret coefficient over the composition space. Figure 1B presents the composition dependence of the Soret coefficient and the Soret vector (shown along the horizontal axis) of the THN–nC12 binary mixture. The Soret coefficient of the THN–nC12 binary mixture is positive over the entire composition range^24^. THN is a heavier component, and in case of a stable Soret separation, it moves to the cold side, so, the blue side of the vector is aimed at increasing the THN content. The length of the Soret vector is proportional to the value of the Soret coefficient.

Another example is the water–ethanol mixture. The Soret coefficients were measured over the entire composition range^25^, and it exhibits the sign changes at the water mass fraction $w_{wat}$ = 0.78 kg/kg, see Fig. 1C. Water is a heavier component affecting the stability of the separation. In the region of the positive Soret sign, water goes to the cold side, and the blue side of the vector is directed towards the increase in water. In the region where the Soret sign is negative, water goes to the hot side, and, thus, the red side of the vector is directed towards the increase in water. The length of the vector characterizes the strength of the Soret coefficient.

We will discuss the benefits of the vector representation of the Soret-driven separation for extrapolating knowledge from binary limits into a ternary system using three ternary mixtures in order of increasing complexity of their behavior: the nearly ideal mixture THN–IBB–nC12 with positive Soret coefficients in all subsystems; the non-ideal TEG–Wat–EtOH mixture with the $S_{T}^{\prime \,bin}$ sign change in two binary subsystems and the Tol–MeOH–Ch mixture with the $S_{T}^{\prime \,bin}$ sign change in two subsystems and an extended miscibility gap. Hereafter, the notation with the superscript “bin” ($S^{\prime \,bin}_{T}$) stands for binary mixtures and without ($S'_{T}$) for ternary mixtures.

### Nearly ideal mixture tetralin–isobutylbenzene–*n*-dodecane (THN-IBB-nC12)

We argue that knowledge of the Soret coefficients of binary subsystems can predict the sign of the ternary Soret coefficients in the entire composition pool, as well as outline areas of sign reversal and identify the areas with the greatest separation and the component that determines this. The predictive role of the vector presentation can be best demonstrated using the THN–IBB–nC12 mixture, as the foreknowledge can be verified by measurements carried out by various research groups.

**Figure 2:**
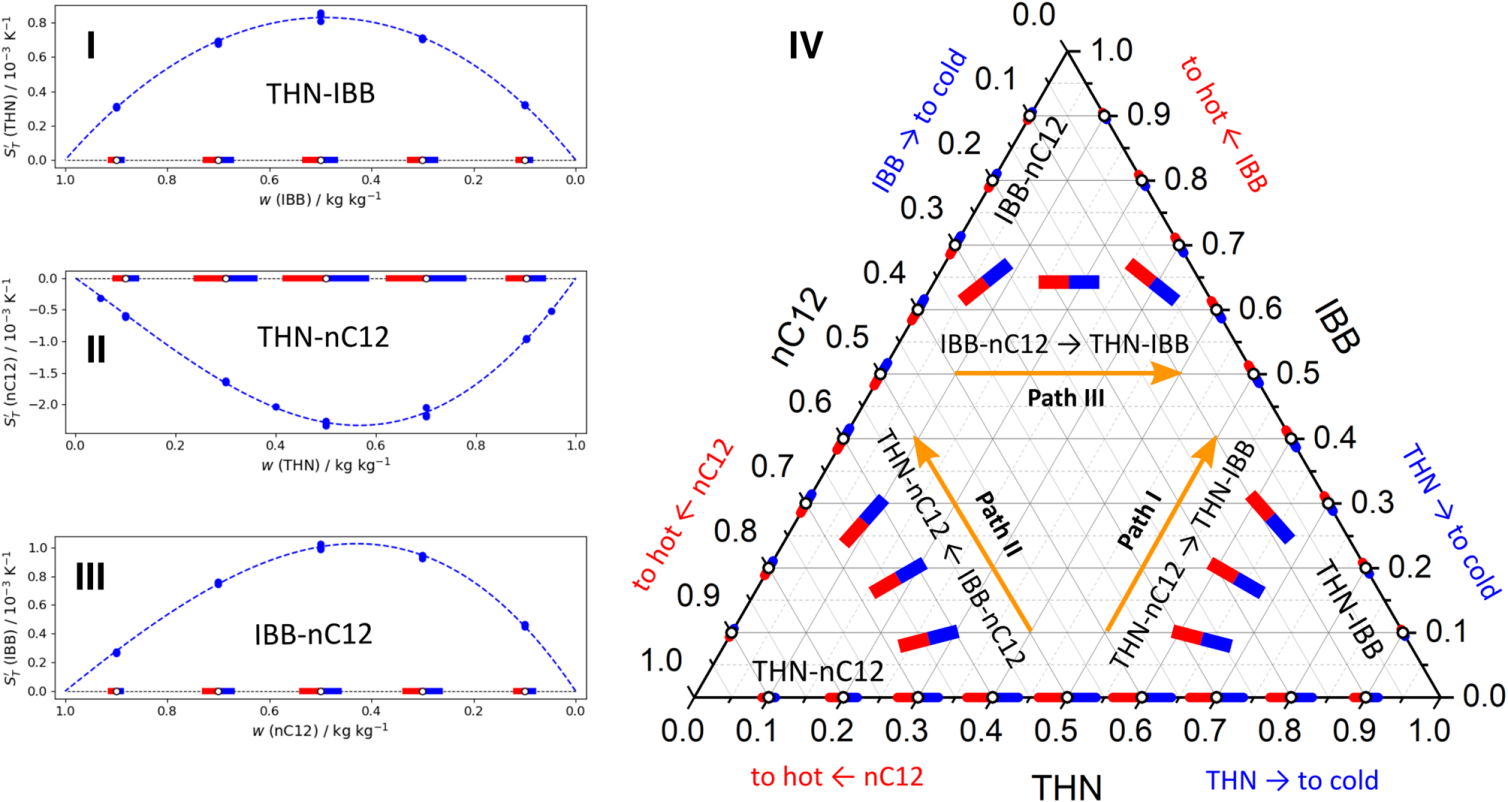
The Soret vectors in binary subsystems of the ternary mixture THN–IBB–nC12 are shown individually, (**I**) THN–IBB; (**II**) THN–nC12; (**III**) IBB–nC12, and on the sides of the Gibbs triangle (in mass fraction units). Paths (**I**–**III**) trace the Soret coefficients of THN, nC12 and IBB, respectively. The vectors on all the sides are plotted according to measurements and using the same scale. White circles indicate to which state point corresponds the vector. The vectors inside a ternary plot without an open circle in the center display the expected orientation and not in scale. The order of components in each binary subsystem corresponds to decreasing density and, for clarity, they are also written in gray letters.

Figure 2 on the left side presents the Soret coefficients and vectors in binary subsystems and combines them on the Gibbs triangle on the right side. We start analysis of the THN–IBB–nC12 ternary mixture by examining the change in the direction of the Soret vector between two binary subsystems including THN: THN–nC12 and THN–IBB. The creation of the Soret vector for the THN–nC12 mixture was illustrated in Fig. 1B. For the THN–IBB mixture, the vectors are displayed in Fig. 2I as a function of the IBB content, to match concentration axes of the triangle. The Soret effect of THN, $S'_T$(THN), can be traced along path I. Let us select the state point with $w_{THN}=0.5$ of the THN–nC12 binary mixture and move along path I (i.e., THN isoline) towards the THN–IBB mixture. On this path, the Soret vector rotates, as sketched by the three vectors in the right corner. At the other end, in the THN–IBB subsystem, it is turned by 60$^{\circ }$ relative to its original position. The binary vectors have a somewhat similar direction, that is, the blue side of the vectors of both subsystems is directed to the lower right corner. Knowing only the binary Soret coefficients, it is difficult to predict how uniform the rotation is. Another observation is that the vectors in THN–nC12 are longer than those on the other side, indicating that the $S'_T$(THN) coefficient significantly decreases approaching THN–IBB mixture.

We suggest that if the blue (or red) sides of binary vectors are aimed at the apex in both subsystems, then the vector turns over the concentration path only by 60$^{\circ }$. Accordingly, the ternary Soret coefficient of the component situated in the vertex does not change sign. Formally, the sign can be changed twice, but to the best of our knowledge, this has not yet been observed in binary mixtures. Therefore, since the binary Soret coefficient is positive at the beginning and end of path I, it remains positive all the way. The same mutual matching of the binary vectors is valid for any parallel isoline between these binary subsystems. Thus, we conclude that the Soret coefficient of THN, $S_{T}^{\prime }$(THN), in the THN–IBB–nC12 ternary mixture is positive along either concentration isoline, that means, in the entire compositions pool.

Next, we will examine the evolution of the Soret vector between the binary subsystems THN–nC12 and IBB–nC12 along path II in Fig. 2IV, with the target to provide a hint for the sign evolution of $S_{T}^{\prime }$(nC12) in the ternary mixture. We choose the state point with $w_{\,nC12}=0.5$ at the bottom of the triangle and move towards the IBB–nC12 mixture. Again, over path II the vector turns by 60$^{\circ }$ and it is also sketched by the three vectors in the left corner. The red sides of the vectors of both subsystems point to the same apex. This suggests that the Soret coefficient does not change the sign along the path. Since in a binary mixture $S^{\prime \,bin}_{T,1}+S^{\prime \,bin}_{T,2}=0$, see Eq. (4), the Soret coefficients of components have opposite signs, e.g.,

$$\begin{aligned} S^{\prime \, bin}_{T}\text {(nC12)}=-S^{\prime \, bin}_{T}\text {(THN)} \quad \text{ and } \quad S^{\prime \, bin}_{T}\text {(nC12)}=-S^{\prime \,bin}_{T}\text {(IBB).} \end{aligned}$$

As an illustrative example, the dependence of $S^{\prime \, bin}_{T}$(nC12) on the mass fraction of THN in the THN–nC12 mixture is shown in Fig. 2II and can be compared with $S^{\prime \, bin}_{T}$(THN) in Fig. 1B for the same mixture. The Soret coefficient of nC12 is negative on both sides and therefore along the path. Since the Soret coefficients in both subsystems do not change sign over the entire range of compositions, $0<w_3<1$, this means that $S_{T}^{\prime}$(nC12) is negative on any parallel path and in the entire ternary mixture.

**Figure 3:**
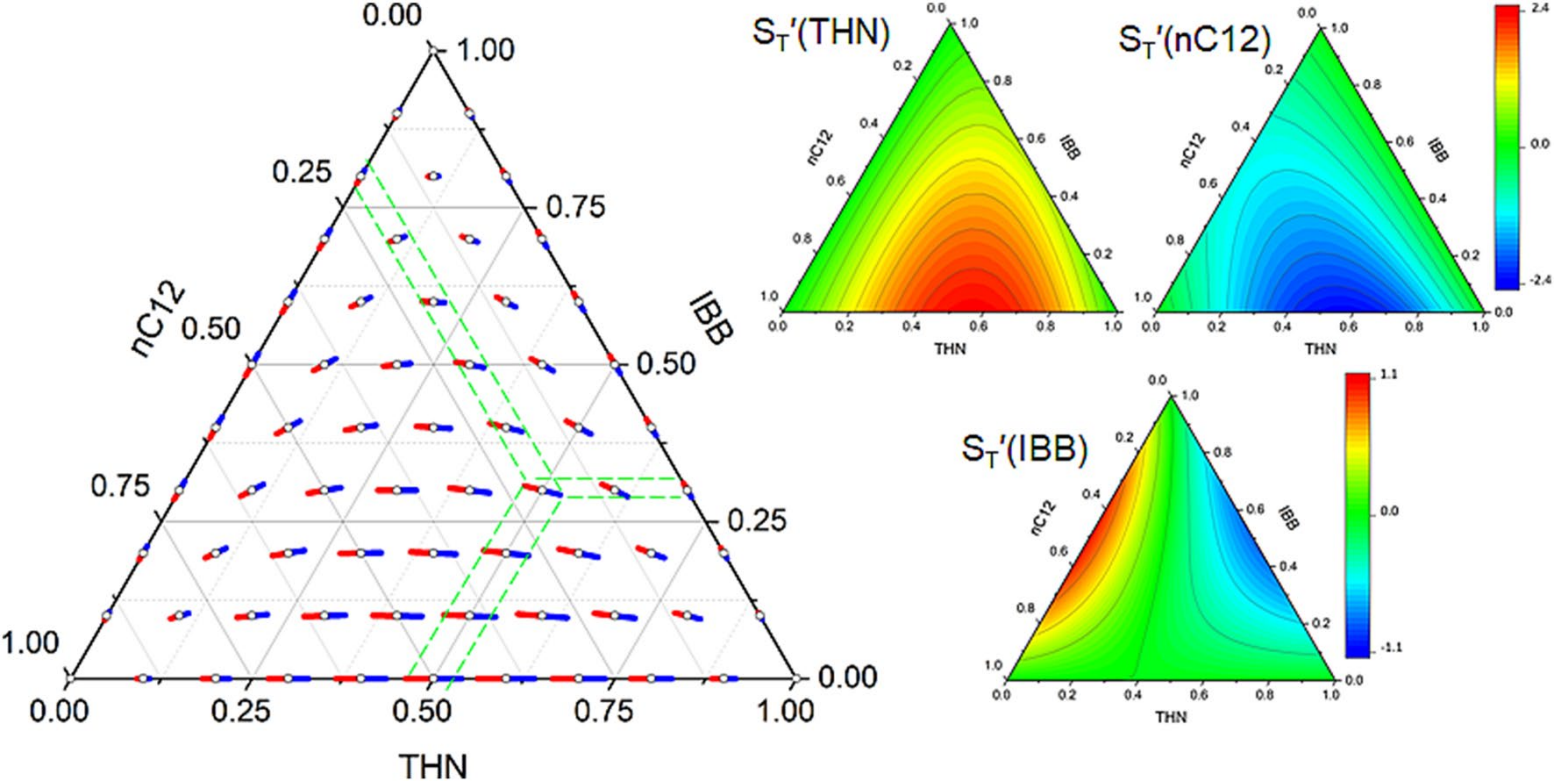
The Soret vector field in the THN–IBB–nC12 ternary mixture offering a valuable overview of the entire Soret separation. The green lines illustrate the setting up the vector projections on the axes. The colored triangles on the right hand side present the distribution of the $S_{T}^{\prime }$(THN), $S_{T}^{\prime }$(IBB), and $S_{T}^{\prime }$(nC12) over the composition space according to Ref.^26^. The color scale for $S_{T}^{\prime }$(THN), $S_{T}^{\prime }$(nC12) is the same and for $S_{T}^{\prime }$(IBB) is halved.

Finally, we will analyze the path between two binary subsystems IBB–nC12 and THN–IBB to consider the Soret sign of the IBB component. The creation of the Soret vectors for this side is illustrated in Fig. 2I,III. Close inspection of the ternary plot reveals that the Soret vectors in IBB–nC12 and THN–IBB show the opposite direction: the blue side of the IBB–nC12 vectors and the red side of the THN–IBB vectors are directed to the vertex. Thus, moving along path III, from the IBB–nC12 to THN–IBB, the Soret vector rotates by $120^{\circ }$ to comply with both boundary conditions. According to our hypothesis, the Soret coefficient also changes sign from positive to negative along the path. This is valid for any other parallel path. The Soret sign in the binary subsystems is positive for nC12-rich and is negative for THN-rich mixtures. Thus, the sign change in the ternary mixture can be predicted considering the Soret vectors of the binary subsystem. It is challenging to predict where sign change occurs. Given that at the upper part, the Soret vectors of IBB–nC12 and THN–IBB mixtures have a similar length on both sides, one can guess that this occurs in the middle of the ternary plot when the contents of THN and nC12 are nearly equal. In the lower part, it could be shifted to the side of higher IBB content.

Now we turn to the verification of the hypothesis that $S_{T}^{\prime }\text {(THN)}>0$, $S_{T}^{\prime }\text {(nC12)}<0$, and $S_{T}^{\prime }$(IBB) changes the sign. For this ternary mixture, the Soret coefficients inside the triangle were measured by several teams^11,14,20,26,27^, and it provides the opportunity to build a complete vector field, which is presented in Fig. 3. The first check shows that all the measurements are consistent, as the vector field does not show abrupt transitions. Second, it identifies the largest separation occurs in the region with a lower content of IBB, less than 50%. Following the same paths as in Fig. 2, one will find that all of the predictions about the sign of $S_{T,i}^{\prime }$ are correct: $S_{T}^{\prime }\text {(THN)}>0$ and $S_{T}^{\prime }$(nC12) does not change sign but $S_{T}^{\prime }$(IBB) does. The projection of the vector field on the isoline with constant content of IBB clearly shows the negative slope on the left side and positive on the right side of the ternary plot. In the central and especially in the lower part, the vector projection for IBB almost disappears, indicating a small value of $S_{T}^{\prime }$(IBB).

For undoubted verification of the above considerations, the experimental results from Ref.^26^ are presented for individual components of the mixture in the form of color-contour plots on the right side in Fig. 3. In complete agreement with our analysis, they show that $S_{T}^{\prime }$(THN) is positive, $S_{T}^{\prime }$(nC12) is negative for all the compositions and $S_{T}^{\prime }$(IBB) changes sign. It is worth noting that the assumption about the locus of points with sign change is also supported by the measurements. As expected, the largest Soret separation occurs in the region where the content of IBB is less than 50%.

**Table 1:** Summary of the Soret coefficients measured in microgravity (ISS)^15^ and by two ground-based techniques OBD (optical beam deflection) and TGC (thermo-gravitational column) from Refs.^15,28^.

Point #	*w*(TEG)	*w*(Wat)	*w*(EtOH)	$S_{T}^{\prime }$(TEG)	$S_{T}^{\prime }$(Wat)	$S_{T}^{\prime }$(EtOH)
1 (OBD)	0.6	0.2	0.2	− 0.7	0.4 ± 0.5	0.3 ± 0.5
2a (OBD)	0.34	0.33	0.33	− 0.42 ± 0.22	0.97±1.02	− 0.55 ± 1.24
2b (TGC)	0.34	0.33	0.33	− 0.35 ± 0.02	0.97 ± 0.04	− 0.62 ± 0.05
2c (ISS)	0.34	0.33	0.33	− 0.26 ± 0.12	1.64 ± 0.51	− 1.38 ± 0.62
3a (OBD)	0.15	0.25	0.6	− 0.3	0.2 ± 0.4	0.1 ± 0.4
3b (TEG)	0.15	0.25	0.6	− 0.31	1.34	− 1.03
4 (OBD)	0.1	0.75	0.15	− 0.1	− 0.4 ± 0.4	0.5 ± 0.1
5 (OBD)	0.4	0.5	0.1	− 0.2	0.7 ± 0.6	− 0.5 ± 1.1

Soret coefficients in units of $(10^{-3} K^{-1})$, content *w* in mass fractions. The error bars are not displayed if they are missing in the original papers.

**Figure 4:**
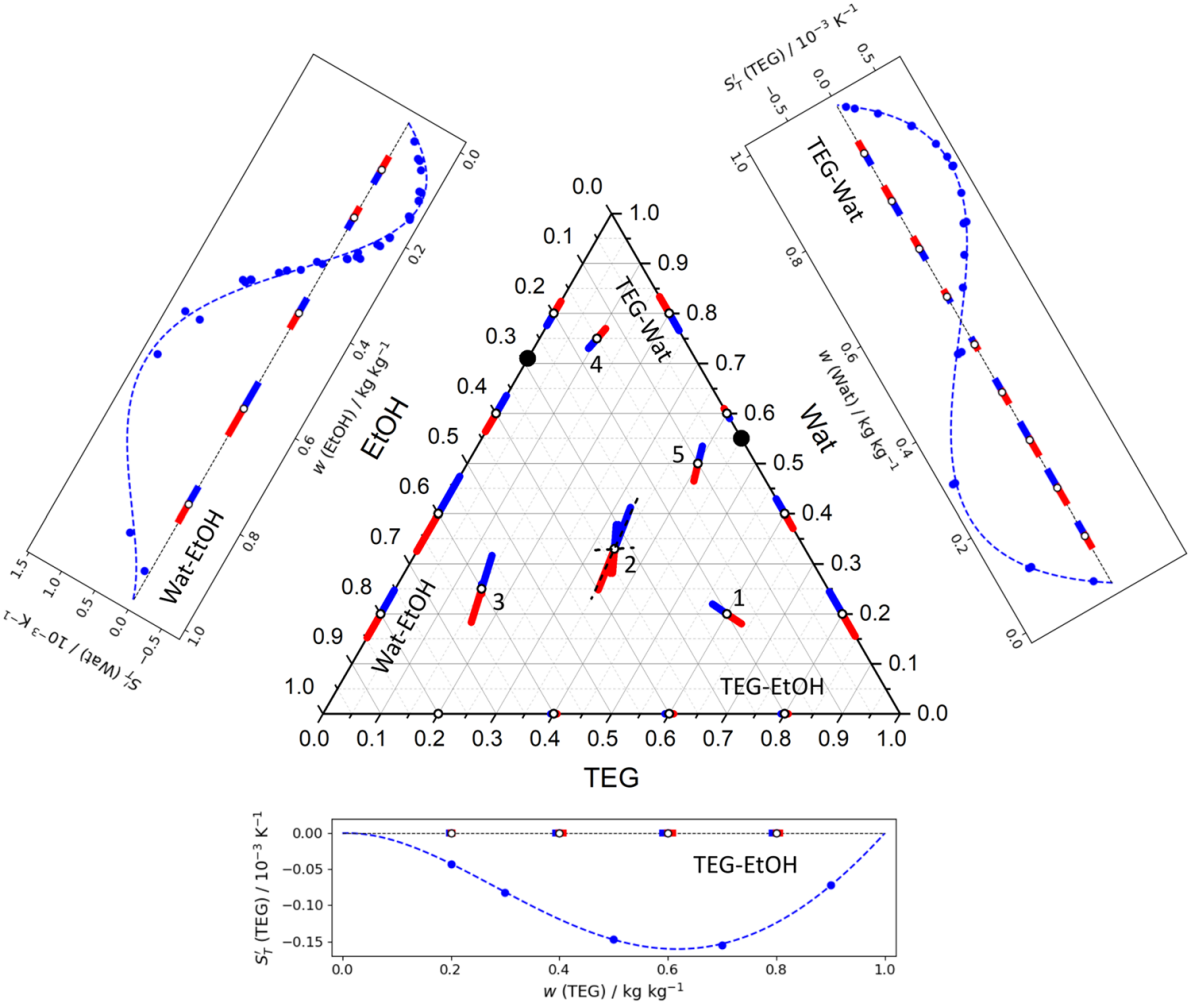
The arrangement of the Soret vectors in binary subsystems and in the TEG–Wat–EtOH ternary mixture at $T=298$ K. It follows that the component separation decreases sharply at low water content. The vectors in the state point #2 reflect three different approaches (see Table 1): the smaller vectors for TGC and OBD coincide, and the larger vector presents the ISS results. The thin black dashed lines at the point #2 show the limiting positions of the vector, covering the interval of error bars. The two black circles indicate the sign change of the Soret effect. Note that the values on the axes for the Wat–EtOH and TEG–Wat mixtures are plotted in reverse order to match the axes of the triangle. The symbols on binaries corresponds to the measured points and the dashed curves are intended as guide for the eye. The values of $S_{T}^{\prime }(i)$ for the ternary mixture are taken from Refs.^15,28^. The Soret coefficients for Wat–EtOH are from Ref.^25^, for TEG–Wat and TEG–EtOH the authors’ unpublished data^29^, as well as the data of W. Köhler’s group (Uni.Bayreuth)^30^.

**Figure 5:**
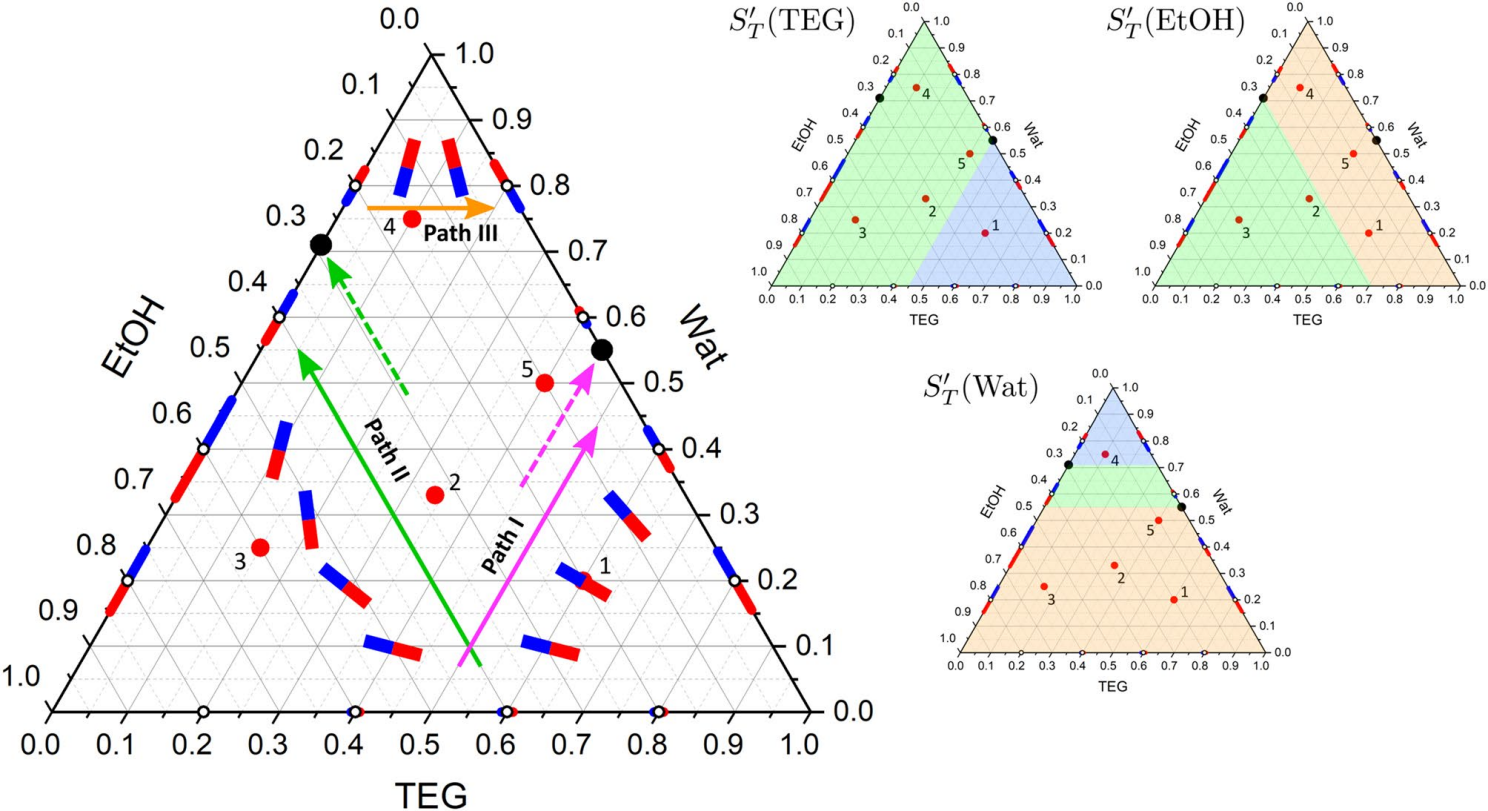
Prediction of the Soret sign in the TEG–Wat–EtOH ternary mixture and consistency check of the experimental results^15,28^. Paths I, II, III trace $S_{T}^{\prime }$ in TEG, EtOH and Water, respectively. The vectors inside a ternary plot without an open circle display the expected orientation and not in scale. The colored triangles illustrate the predicted Soret sign in the mixture: the blue region corresponds to the negative sign, the beige—to the positive sign, and the green region allows a sign change within the area.

### Non-ideal mixture triethylene glycol–water–ethanol (TEG–Wat–EtOH)

Experiments with TEG–Wat and Wat–EtOH mixtures are challenging due to large regions with negative Soret effect. As a consequence, the transport coefficients have been measured quite recently and have not even been published in full. The only known values of the coefficients for five ternary solutions are given in Table 1. Note that state point #2 was measured by two ground techniques and on the ISS^15,28^. For all the measured systems, Fig. 4 combines binary and ternary Soret vectors. It is more complicated to arrange vectors on the Gibbs triangle for this mixture since two subsystems change sign^30^. For clarity, the Soret coefficients in binary subsystems are placed next to their location on the ternary plot with the values on the axes in reverse order. The first inspection indicates that the Soret separation is very weak in the water-poor region. Another observation is that at point #2 various experimental approaches provide vectors with slightly different directions. The vectors of ground measurements (OBD and TGC) overlap, and results from the ISS correspond to a larger vector with a slightly different direction.

It is more convenient to examine the Soret sign in this ternary system using simultaneously Figs. 4 and 5. We start from the TEG sign, following path I from TEG-EtOH to TEG-Wat mixture. The Soret effect for TEG-EtOH is negative over the full composition range while TEG-Wat changes sign at $w_{TEG}=0.45$ kg/kg as clearly seen in the binary plots in Fig. 4. For TEG-rich part of the mixture ($w_{TEG}>0.45$ kg/kg), the red side of the vectors in both subsystems is directed to the apex. Since $S_{T}^{\prime \,bin}\text {(TEG)}<0$ in both subsystems, it suggests that $S_{T}^{\prime }$(TEG) is also negative for this composition pool which shown by the blue shading in the separate plot for $S_{T}^{\prime }$(TEG) in Fig. 5. As soon as TEG-Wat changes the Soret sign, the binary vectors start pointing in the opposite directions, that means the ternary $S_{T}^{\prime }$(TEG) will also change sign from negative to positive with increasing water content (somewhere within the green shading).

To examine the Soret sign of EtOH, the rotation of the Soret vector between the TEG–EtOH and Wat-EtOH subsystems is investigated when moving along path II. As on path I, one mixture (TEG–EtOH) does not change sign, while the other (Wat—EtOH) changes at $w_{EtOH}$=0.3 kg/kg. It can be seen in Fig. 5 that in the ethanol-rich mixture, ($w_{EtOH}>0.3$ kg/kg), different sides of the Soret vectors of two subsystems point to the left corner, i.e., the vector rotated by $120^{\circ }$. This suggests that $S_{T}^{\prime }$(EtOH) changes sign along path II and on any isolines when $w_{EtOH}>0.3$ kg/kg. The large region where the sign change occurs is green-shaded in the separate plot for $S_{T}^{\prime }$(EtOH) in Fig. 5. On the contrary, for a mixture with a low EtOH content, the Soret sign of EtOH in Wat–EtOH mixture is positive. The binary vectors of two subsystems in this concentration space ($w_{EtOH}<0.3$ kg/kg) are turned by $60^{\circ }$ to each other, which assumes that $S_{T}^{\prime }$(EtOH) does not change sign. This suggests that $S_{T}^{\prime }$(EtOH) is positive in the region which is shaded in beige in the $S_{T}^{\prime }\text {(EtOH)}>0$ plot in Fig. 5. Recall that the signs of binary components are opposite, see Eq. (4). However, we should be more careful with this statement about the point #5, since it is located in the vicinity of the singular point and the attractor can change the situation in some way.

Tracking path III and its parallel routes include two singular points, one on the Wat–EtOH side and the other on the TEG–Wat side, as shown by the black circles in Fig. 5. As a result, the ternary plot for $S_{T}^{\prime }$(Wat) consists of three regions: a larger one with a positive sign (beige-shaded in the separate ternary plot), a smaller one near the upper apex with a negative sign (blue-shaded) and a zone between two singular points (green-shaded) where $S_{T}^{\prime }$(Wat) can be positive or negative.

In order to compare the analysis above with the measurements presented in Table 1, the experimental points will be discussed one by one. It follows from Fig. 5 that for point #1 the signs for all $S_{T}^{\prime }$ are unambiguously predicted, and they completely coincide with the measured ones. For point #2, the positive sign is clearly identified for $S_{T}^{\prime }$(Wat), and it is in agreement with measurements. For $S_{T}^{\prime }$(EtOH), the measurements provide a negative sign. Since this point on the $S_{T}^{\prime }$(EtOH) ternary plot is close to the region with a positive sign, it is expected that the coefficient value should not be large.

For addressing the errors in determining the ternary Soret coefficients and their potential impact on the vector’s norm and orientation, point #2 is a good example. This state point was measured thoroughly, and hence characterised in a more complete way than others. The measurements were collected from three different instruments, and error-bars were assessed for each of them^15^. The error bars for each technique primarily depend upon the condition number of the contrast factors matrix. To enhance visibility, we have chosen and drawn the highest error-bar among the data presented in the reference. The limiting positions of vector, enclosing the span of the error-bars at point #2, are shown in Fig. 4 by the thin black dashed lines. They form an angular interval of allowed orientations of the Soret vector. The figure shows that all individual vectors measured at this state point do fall into the span, although the ISS data are located at its very limit. This example is indicative, thanks to its unusually large errors that strongly affect both the norm and the orientation of the vector. This results from a poor mutual orientation of the Soret vector and the refractive index isolines with an acute angle between them, as well as due to the very high condition number of the matrix of optical contrast factors^31^. From the interval limited by the error-bars at this point, it follows that the small or even slightly positive $S_{T}^{\prime }$(EtOH) is still a viable option. We did not discuss the error bar for the hydrocarbon mixture above, since the vectors were plotted there after a uniform parametrization and not after individual measurements, hence with the error eliminated.

At point #3, $S_{T}^{\prime }\text {(Wat)}>0$ and this is in line with expectations and experiments. As concerns $S_{T}^{\prime }$(EtOH), different techniques provided opposite signs, and from our analysis both signs are possible (the green region in Fig. 5). However, the ternary vector in Fig. 4 is almost parallel to the vectors for the Wat–EtOH mixture where $S_{T}^{\prime \,bin}$(EtOH) is negative, and we expect the sign of the ternary mixture also to be negative. The measurements and predictions for the Soret signs at state point #4 are in complete agreement.

However, there is some discordance at point #5 for $S_{T}^{\prime }$(EtOH) which lies deeply in the positive region in the ternary plot in Fig. 5 but measurement provides a negative sign. Figure 4 also reveals some controversy in the evolution of the vectors between points 4, 5 and 1. Note that the state point #5 is located near the singular binary point and $S_{T}^{\prime }$(Wat), $S_{T}^{\prime }$(TEG) at this point are very close to the border with the sign change. This gives a hint that the proposed analysis may not be applicable near the singular point and requires deeper attention. Nevertheless, this analysis shows that the vector consideration can identify the peculiarity of experimental results.

### Mixture toluene–methanol–cyclohexane (Tol–MeOH–Ch) with extended demixing zone

**Figure 6:**
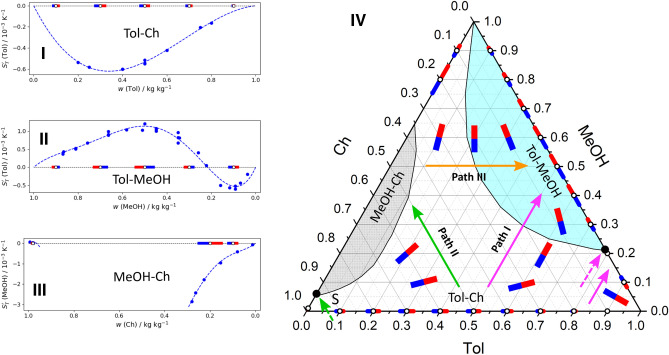
The Soret vectors in binary subsystems of the ternary mixture Tol–MeOH–Ch are shown individually, (**I**) Tol–Ch, (**II**) Tol–MeOH, (**III**) MeOH–Ch, and on the sides of the Gibbs triangle (**IV**). Paths (I–III) trace the Soret coefficients of Tol, Ch and MeOH, respectively. The known vectors on all the sides are shown using the same scale, while the vectors inside a ternary plot display an expected orientation and are not in scale. The demixing zone is shaded in gray and the stable stratification region is shaded in blue.

This mixture is more complex because, besides a sign change of the Soret effect in two binary subsystems, it contains a demixing zone. The binary vectors of these mixtures are shown separately and on the ternary plot in Fig. 6.

The MeOH–Ch mixture, which exhibits a miscibility gap at ambient conditions, was measured back in 1969 by Story and Turner^32^. Except for a very small cyclohexane-rich area below the demixing zone, $S_{T}^{\prime \,bin}\text {(MeOH)}<0$. Since the heavier component has a negative Soret sign, the components separation leads to gravitational instability, which imposes experimental difficulties. Another binary subsystem, the Tol–Ch mixture, which was measured on ground^33,34^ and onboard the ISS^34^, shows a negative Soret coefficient throughout the composition range. Only Tol–MeOH mixture has a large region with positive Soret coefficients. The overall picture from the Gibbs triangle is that the Soret separation significantly different over the concentration space.

Some properties of the Tol–MeOH–Ch mixture have been extensively studied in the ground laboratory since it was a candidate for testing on the ISS^18^. The target of the pioneer ground tests^35^ was to determine the region of gravitational stability of the mixture under a thermal gradient to facilitate a further detailed study of transport properties. The region with stable Soret separation is illustrated by the blue shading on the ternary plot in Fig. 6.

As in the previous cases, the mutual location of binary vectors may suggest the Soret sign of the ternary mixture. Here it is more convenient to begin consideration with the sign of cyclohexane, i.e., following path II. Between Tol–Ch and MeOH–Ch (above the miscibility gap) mixtures, the Soret vector turns by $60^{\circ }$, the red sides of the vectors are aimed at the Tol–MeOH side. In both subsystems, cyclohexane is the lighter component and $S_{T}^{\prime \,bin}$(Ch) is positive. Apparently, the Soret sign of $S_{T}^{\prime }$(Ch) is positive everywhere in the ternary space except the tiny zone below the miscibility gap where MeOH–Ch changes the sign (marked with point S).

The sign of $S_{T}^{\prime }$(MeOH) is examined considering the rotation of the vector along path III, between MeOH–Ch and Tol–MeOH mixtures. The whole space can be separated in three sectors: the largest one including the stability region where $w_{MeOH}>0.22$ kg/kg, the part between two singular points, $w_S<w_{MeOH}<0.22$ kg/kg (the content of MeOH corresponding to point *S* is not known exactly), and the small part near the bottom where $0<w_{MeOH}<w_S$.

At the largest upper part, the red sides of vectors in both subsystems point out the apex indicating the vector turn by $60^{\circ }$. Since MeOH has a negative Soret sign in both subsystems, it remains negative in the ternary space. In the sector between two singular points, the Soret effect may change sign. At the lowest small part (below the miscibility gap), the red sides of the vectors again are coordinated, and aimed at the bottom. As on both sides $S_{T}^{\prime }$(MeOH) is positive, it is also positive in this sector.

Finally, we return to path I following between the Tol–Ch and Tol–MeOH mixtures. In the Tol-rich corner, the red sides of both vectors are directed to the apex assuming that the ternary Soret coefficient of toluene does not change sign until it reaches a singular point at $w_{Tol}=0.78$ kg/kg. Given that $S_{T}^{\prime \,bin}$(Tol) is negative on both sides, then $S_{T}^{\prime }\text {(Tol)}<0$ near the corner. At the lower Tol content, path I traverses the stability boarder. According to the different directions of the Soret vectors at Tol–Ch and Tol–MeOH mixtures, it is expected that $S_{T}^{\prime }$(Tol) changes sign. To conclude, $S_{T}^{\prime }\text {(Ch)}>0$ and $S_{T}^{\prime }\text {(MeOH)}<0$ except a small area below the miscibility gap, and $S_{T}^{\prime }\text {(Tol)}>0$ at the stable region, otherwise it is negative.

### Associating the Soret vector with density and refractive index maps

Soret coefficients in the ternary Tol–MeOH–Ch mixture have been less studied by direct measurements in comparison with the two previous systems. There is one single measurement available in literature for the state point with composition 0.62/0.31/0.07 in mass fractions^18^. Besides that, a detailed analysis of hydrodynamic stability of the ternary Soret separation is available^35^. These data, supplemented with a knowledge on refractive index^36^ and density^12^ of the mixture over the entire concentration range, allow validating the prediction from another perspective.

Modern methods for studying ternary mixtures under a temperature gradient use either a change in the refractive index (*n*) at two different wavelengths, or a change in the refractive index (*n*) and density ($\rho$). The separation created by the Soret effect can be well tracked by variations of these properties, whose values are different on cold and hot sides. Both properties depend upon temperature and composition, but here we consider only their change due to the Soret effect in the form of concentration-induced variation at steady-state, normalised by $\Delta T$.

**Figure 7:**
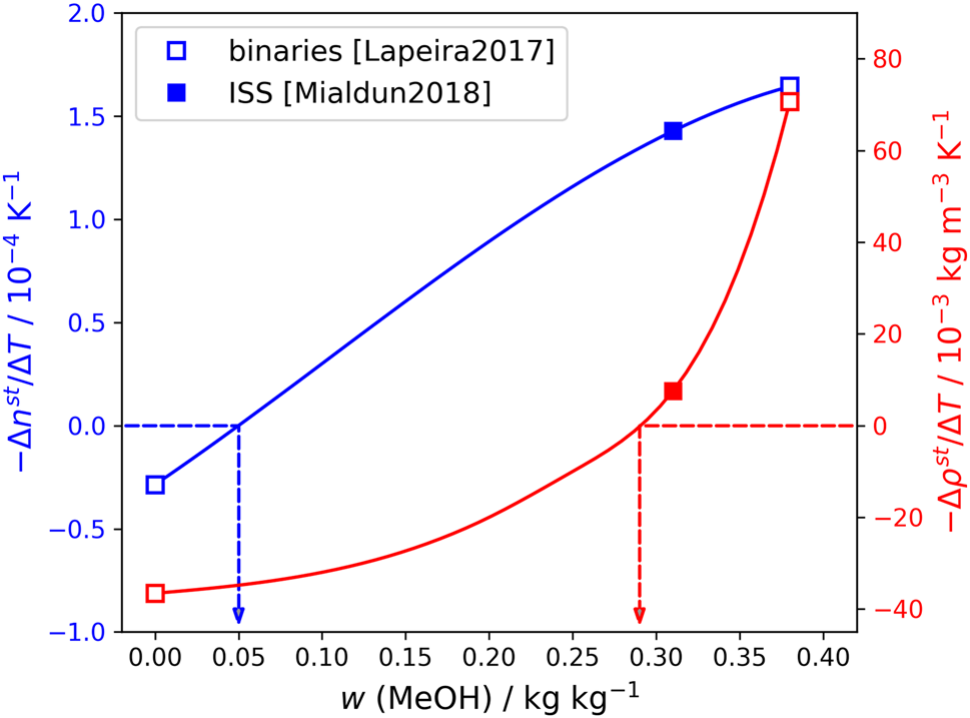
The separation in the ternary mixture Tol–MeOH–Ch along the path with toluene constant content, $w_{Tol}=0.62$ kg/kg, characterised by refractive index $-\Delta n^{\text{ st }}/ \Delta T$ (blue, left axis) and by density $-\Delta \rho ^{\text{ st }}/ \Delta T$ (red, right axis).

The observable separation is determined as a projection of the Soret vector on the gradient of density or the refractive index. Mathematically, it can be expressed as the dot product of the two vectors

6 $$\begin{aligned} -\frac{\Delta \rho ^{\text {st}}}{\Delta T} = \pmb {\nabla \rho } \cdot \pmb {S'_T}, \qquad \qquad -\frac{\Delta n^{\text {st}}}{\Delta T} = \pmb {\nabla n} \cdot \pmb {S'_T}, \end{aligned}$$

where the density and refractive index gradients are defined as

7 $$\begin{aligned} \pmb {\nabla \rho } = \left( \frac{\partial \rho }{\partial w_1} \right) \pmb {e_1} + \left( \frac{\partial \rho }{\partial w_2} \right) \pmb {e_2}, \qquad \pmb {\nabla n} = \left( \frac{\partial n}{\partial w_1} \right) \pmb {e_1} + \left( \frac{\partial n}{\partial w_2} \right) \pmb {e_2}. \end{aligned}$$

Here, the equivalence of the dot product and the scalar projection arises due to the fact that all concentration derivatives of the properties are obtained on the unit concentration space.

To better understand how a change in property caused by the separation may help in clarifying the orientation of the Soret vector, we consider its behaviour along a path, similar to path I in Fig. 6. We choose the path that follows the isoline $w_{Tol}=0.62$ kg/kg, since there are three measured points on the path: two binary limits^37^ and one ternary point^18^. Soret coefficients at these points were recalculated to the quantities $-\Delta \rho ^{\text {st}}/ \Delta T$ and $-\Delta n^{\text {st}}/ \Delta T$ using data from above references and Eqs. (5), (6) and (7). Both quantities, shown in Fig. 7 versus methanol concentration, do vanish at certain points on the path. This vanishing does not mean that the Soret separation disappears at these points, it simply means that the presence of the particular property’s variation cannot be detected there. Equation (6) relates this situation to the orientation of the Soret vector when it becomes normal to the gradient of the property or, alternatively, when it becomes parallel to the isoline of the property.

Because in the Tol–MeOH–Ch mixture the behavior of the refractive index and density is not identical, the points with optically vanishing separation and iso-dense separation are located at different compositions; the former corresponds to $w_{MeOH}=0.05$ kg/kg, while the latter is about $w_{MeOH}=0.28$ kg/kg (cf. annotation arrows in Fig. 7). It is worth noting, that the location of these zero points is approximate due to scarce data points.

**Figure 8:**
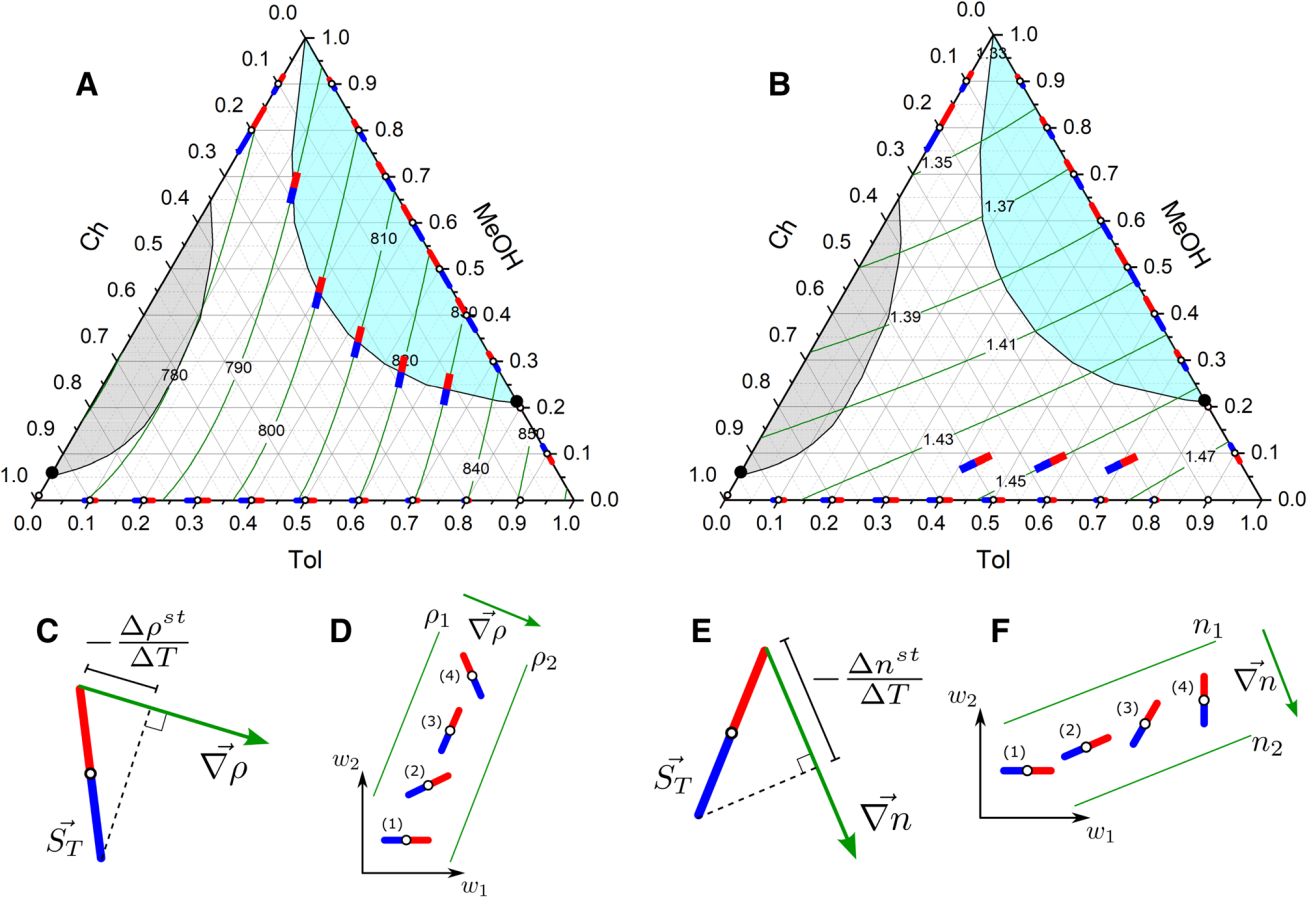
Binding the Soret vector with the density (**A**) and refractive index (**B**) maps in the Tol–MeOH–Ch ternary mixture. The green lines illustrate the isoline of either $\rho$ (**A**) or *n* (**B**). The demixing zone is shaded in gray and stability region is shaded in blue. The Soret vectors, parallel to the density isolines near the stability boundary in (**A**), illustrate the situation when separation occurs, but the density does not change over the cell (isodense separation). The Soret vectors inside (**B**) illustrate the situation when separation occurs, but the refractive index does not change across the cell. (**C**,**E**) Illustrate graphically the projection of the Soret vector onto the gradient of density (**C**) or refractive index (**E**). Sketch of the Soret vector rotation in the space between two binary subsystems, which, in turn, changes its position relative to the density isoline (**D**) or refractive index (**F**) (vectors are not in scale).

The maps of density and refractive index are superimposed on binary vector fields in Fig. 8A,B where green lines are isolines of the property. Two panels, (C,E), present the graphic equivalent of Eq. (6). Two other panels, (D,F), sketch the change in the projections of the Soret vector in space between the two binary subsystems (Tol–Ch and Tol–MeOH) when $w_{Tol}<0.78$kg/kg and its orientation with respect to the property. Drawing up projections in ternary plot was illustrated in Fig. 3 for all the components.

First, let us correlate the Soret separation with the density. Figure 8D outlines the rotation of the Soret vector relative to the density isolines along the path, which approximately corresponds to that one discussed in Fig. 7. The orientation of the Soret vectors at points (1) and (2) results in negative values of $(-\Delta \rho ^{\text {st}}/\Delta T)$ or, in other words, in unstable density stratification. At point (3) the quantity vanishes, $(-\Delta \rho ^{\text {st}}/\Delta T)=0$. This occurs when the Soret vector is parallel to the density isoline, which indicates that at this composition, the density of the ternary mixture on the cold and hot sides is the same. This arrangement matches with the neutral stability boundary, while at the point (4) the quantity becomes positive and separation is stable.

Figure 7 provides that the Soret separation is iso-dense at a composition $w\approx$ 0.62/0.28/0.10 kg/kg. This value is close to the experimentally found boundary of the stability region (the margin of the blue area). A slight mismatch can be explained by the above mentioned inaccuracy in the localization of the point in Fig. 7 and some uncertainty in mapping the experimental stability boundary. The formal identity of the experimental stability boundary and the iso-dense separation along this path allows to assert with good accuracy that the Soret vector is parallel to the density isolines along the entire length of the stability boundary. The vectors on the boundary of the blue area in Fig. 8A illustrate this statement.

Let us consider in more detail the neutral stability boundary. The overall density difference includes two contributions

$$\begin{aligned} -\frac{\Delta \rho ^{\text {st}}}{\Delta T} = \left( \frac{\partial \rho }{\partial w_1} \right) S'_{T,1} + \left( \frac{\partial \rho }{\partial w_2} \right) S'_{T,2}. \end{aligned}$$

Both density derivatives here are positive, but since $S_{T,2}^{\prime }\text {(MeOH)}<0$, its separation plays against the stability. Thus, the stabilisation occurs due to toluene, which has positive, though small, Soret coefficient; the full density compensation is achieved due to the larger density derivative of toluene. It also signifies that $S_{T,1}^{\prime }$(Tol) has to change sign just below the boundary.

Next, we consider the Soret separation, as it is sensed by refractive index. In the same manner as for density, when turning from one binary boundary to another, the vector has to pass through the orientation where $(-\Delta n^{\text {st}}/\Delta T)=0$. Schematically, this corresponds to point (2) in Fig. 8F. Due to different slopes of the density and refractive index contours, this occurs at different location on Gibbs triangle. In the case of the refractive index, the exact location of zero projection on the path with $w_{Tol}=0.62$ kg/kg also can be obtained from Fig. 7, and it is $w=~$0.62/0.05/0.32 kg/kg. A similar analysis holds for all isolines (paths) of toluene concentration in the range $0<w_{Tol}<0.78$. Figure 8B demonstrates on the Gibbs triangle that when the Soret vector rotates from a horizontal position (at Tol–Ch mixture) to almost a vertical position at the stability boundary, it traverses a position parallel to isolines of *n*. Surely, when the vector is parallel to an isoline, it has zero projection on the refractive index gradient. It occurs when the mass fraction of methanol is in the range $w_{MeOH} = 0.05\ldots 0.10$ all along that span of toluene concentration. The projections of this vector onto the toluene and methanol sides are nonzero, which indicates the existence of the Soret separation, which is not detectable by optical technique.

From the proposed representation of the Soret vectors in Gibbs triangles with density and refractive index maps in Fig. 8A,B, we can conclude that the prediction made on the basis of the Soret vectors on the binary boundaries of the system is fully confirmed by this independent analysis.

## Conclusions

The present study introduces a novel concept, the Soret vector, to elucidate the essential features of Soret driven separation in ternary mixture as a whole. The objective of the study was not only to apply this concept to the most studied ternary liquid mixtures, rather the emphasis lied on understanding common features and how they can be related to the behavior of the binary subsystems. Particularly, the analysis focused on predicting the sign of the Soret coefficients in ternary mixtures, recognizing areas where the separation is higher or lower, visual consistency check of all the data, and associating the Soret vector field with a property map such as refractive index or density.

Three ternary mixtures have been considered in order of the complexity: THN–IBB–nC12, TEG–Wat–EtOH and Tol–MeOH–Ch. Using almost ideal mixture, THN–IBB–nC12, we have presented a methodology for relocating knowledge about the Soret effect in binary subsystems to ternary mixtures. Thanks to numerous measurements and their parametrization, the vector field in the entire ternary mixtures has been constructed and successfully compared with the predictions in terms of the sign and the magnitude of the ternary Soret coefficients. The non-ideal TEG–Wat–EtOH mixture turned out to be a suitable example for considering the Soret sign in a ternary mixture, when the binary subsystems contain two singular points (sign change). In this case, the way of Soret sign prediction is more complicated and requires attention to the order of components on each side of the ternary plot. For the Soret coefficient of each component of the mixture, concentration regions were determined in which the Soret sign is either strictly positive, either strictly negative, or can change sign. The consistency between our predictions and the few available experimental results scattered over the concentration space has been evaluated.

At the next step, the Tol–MeOH–Ch mixture with an extended demixing zone and changeable signs of the Soret coefficient in binary subsystems was investigated. In addition to analysis of the ternary Soret sign, we suggested an insight into association of the vector field with the map of density and refractive index. The projection of the Soret vector onto the density map permits to delineate the region of hydrodynamic stability. In turn, the projection of the Soret vector onto the refractive index map helps to outline the region where Soret separation occurs but is invisible to an optical technique in the steady state.

The results of this study are undoubtedly useful for future measurements of ternary mixtures. The innovative notion of Soret vector is a powerful and flexible tool for analysis of Soret phenomena with more clarity and insight. Thanks to its evident benefit, this new approach deserves more extensive future research on larger series of ternary mixtures.

## References

[1] Galliero G (2016). Impact of thermodiffusion on the initial vertical distribution of species in hydrocarbon reservoirs. Microgravity Sci. Technol..

[2] Niether D, Wiegand S (2019). Thermophoresis of biological and biocompatible compounds in aqueous solution. J. Phys. Condens. Matter.

[3] de Gans B-J, Kita R, Müller B, Wiegand S (2003). Negative thermodiffusion of polymers and colloids in solvent mixtures. J. Chem. Phys..

[4] Würger A (2010). Thermal non-equilibrium transport in colloids. Rep. Prog. Phys..

[5] Zapf D, Köhler W (2020). Thermal and solutal non-equilibrium fluctuations in a polymer solution. J. Chem. Phys..

[6] Errarte A (2020). Thermophoresis as a technique for separation of nanoparticle species in microfluidic devices. Int. J. Therm. Sci..

[7] Bresme F, Hafskjold B, Wold I (1996). Nonequilibrium molecular dynamics study of heat conduction in ionic systems. J. Phys. Chem..

[8] Köhler W, Morozov K (2016). The Soret effect in liquid mixtures—A review. J. Non-Equilib. Thermodyn..

[9] Platten JK (2005). The Soret effect: A review of recent experimental results. ASME. J. Appl. Mech.

[10] Mialdun A, Legros J-C, Yasnou V, Sechenyh V, Shevtsova V (2015). Contribution to the benchmark for ternary mixtures: Measurement of the Soret, diffusion and thermodiffusion coefficients in the ternary mixture thn/ibb/nc12 with 0.8/0.1/0.1 mass fractions in ground and orbital laboratories. Eur. Phys. J. E.

[11] Gebhardt M, Köhler W (2015). What can be learned from optical two-color diffusion and thermodiffusion experiments on ternary fluid mixtures?. J. Chem. Phys..

[12] Kozlova S (2019). Do ternary liquid mixtures exhibit negative main Fick diffusion coefficients?. Phys. Chem. Chem. Phys..

[13] Leahy-Dios A, Bou-Ali MM, Platten JK, Firoozabadi A (2005). Measurements of molecular and thermal diffusion coefficients in ternary mixtures. J. Chem. Phys..

[14] Königer A, Wunderlich H, Köhler W (2010). Measurement of diffusion and thermal diffusion in ternary fluid mixtures using a two-color optical beam deflection technique. J. Chem. Phys..

[15] Triller T (2019). The Soret effect in ternary mixtures of water+ethanol+triethylene glycol of equal mass fractions: Ground and microgravity experiments. Eur. Phys. J. E.

[16] Alonso de Mezquia D (2015). Contribution to thermodiffusion coefficient measurements in DCMIX project. Int. J. Thermal Sci..

[17] Shevtsova V (2015). Dynamics of a binary mixture subjected to a temperature gradient and oscillatory forcing. J. Fluid Mech..

[18] Mialdun A, Ryzhkov I, Khlybov O, Lyubimova T, Shevtsova V (2018). Measurement of Soret coefficients in a ternary mixture of toluene–methanol–cyclohexane in convection-free environment. J. Chem. Phys..

[19] Triller T (2018). Thermodiffusion in ternary mixtures of water/ethanol/triethylene glycol: First report on the DCMIX3-experiments performed on the International Space Station. Microgravity Sci. Technol..

[20] Galand Q (2019). Results of the DCMIX1 experiment on measurement of Soret coefficients in ternary mixtures of hydrocarbons under microgravity conditions on the ISS. J. Chem. Phys..

[21] Mialdun A (2019). Preliminary analysis of diffusion coefficient measurements in ternary mixtures 4 (DCMIX4) experiment on-board the International Space Station. Eur. Phys. J. E.

[22] Vailati A (2020). Giant fluctuations induced by thermal diffusion in complex liquids. Microgravity Sci. Technol..

[23] Bou-Ali MM (2015). Benchmark DCMIX1: Soret, thermodiffusion and molecular diffusion coefficients of the ternary mixture THN–IBB–nC12. Eur. Phys. J. E.

[24] Gebhardt M, Köhler W, Mialdun A, Yasnou V, Shevtsova V (2013). Diffusion, thermal diffusion, and Soret coefficients and optical contrast factors of the binary mixtures of dodecane, isobutylbenzene, and 1,2,3,4-tetrahydronaphthalene. J. Chem. Phys..

[25] Königer A, Meier B, Köhler W (2009). Measurement of the Soret, diffusion, and thermal diffusion coefficients of three binary organic benchmark mixtures and of ethanol-water mixtures using a beam deflection technique. Philos. Mag..

[26] Gebhardt M, Köhler W (2015). Soret, thermodiffusion, and mean diffusion coefficients of the ternary mixture n-dodecane+isobutylbenzene+1,2,3,4-tetrahydronaphthalene. J. Chem. Phys..

[27] Larrañaga M (2015). Contribution to the benchmark for ternary mixtures: Determination of Soret coefficients by the thermogravitational and the sliding symmetric tubes techniques. Eur. Phys. J. E.

[28] Triller, T. *Diffusive Properties of the System Water/Ethanol/Triethylene Glycol in Microgravity and Ground Conditions*. Ph.D. thesis, Bayreuth University (2018).

[29] Santos, C., Barros, M., Ribeiro, A., Mialdun, A. & Shevtsova, V. Investigation of the Soret coefficient in liquid mixtures: TEG–water. In *14th Int. Meeting on Thermodiffusion, Trondheim, Norway*. https://www.ntnu.edu/imt14/programme. Accessed 24 Aug 2021 (2021).

[30] Schraml, M. *et al.* Sign changes of the Soret coefficient in a strongly interacting system: The DCMIX3-system water/ethanol/triethylene glycol. In *14th Int. Meeting on Thermodiffusion, Trondheim, Norway*. https://www.ntnu.edu/imt14/programme. Accessed 24 Aug 2021 (2021).

[31] Shevtsova V, Sechenyh V, Nepomnyashchy A, Legros J (2011). Analysis of the application of optical two-wavelength techniques to measurement of the Soret coefficients in ternary mixtures. Philos. Mag..

[32] Story MJ, Turner JCR (1969). Flow-cell studies of thermal diffusion in liquids. Ppart 5. Binary mixtures of CH3OH with CCl4, benzene and cyclohexane at 25 °C. Trans. Faraday Soc..

[33] Wittko G, Köhler W (2005). Universal isotope effect in thermal diffusion of mixtures containing cyclohexane and cyclohexane-$$d_{12}$$. J. Chem. Phys..

[34] Mialdun A, Shevtsova V (2015). Temperature dependence of Soret and diffusion coefficients for toluene–cyclohexane mixture measured in convection-free environment. J. Chem. Phys..

[35] Shevtsova V, Santos C, Sechenyh V, Legros J-C, Mialdun A (2014). Diffusion and Soret in ternary mixtures. Preparation of the DCMIX2 experiment on the ISS. Microgravity Sci. Tec..

[36] Sechenyh V, Legros JC, Shevtsova V (2012). Measurements of optical properties in binary and ternary mixtures containing cyclohexane, toluene, and methanol. J. Chem. Eng. Data.

[37] Lapeira E (2017). Transport properties of the binary mixtures of the three organic liquids toluene, methanol, and cyclohexane. J. Chem. Phys..

